# Cardio-Protection of Salvianolic Acid B through Inhibition of Apoptosis Network

**DOI:** 10.1371/journal.pone.0024036

**Published:** 2011-09-06

**Authors:** Lingling Xu, Yanping Deng, Lixin Feng, Defang Li, Xiaoyan Chen, Chao Ma, Xuan Liu, Jun Yin, Min Yang, Fukang Teng, Wanying Wu, Shuhong Guan, Baohong Jiang, Dean Guo

**Affiliations:** 1 Shanghai Institute of Materia Medica, Chinese Academy of Sciences, Shanghai, China; 2 Shenyang Pharmaceutical University, Shenyang, China; Istituto Dermopatico dell'Immacolata, Italy

## Abstract

Targeting cellular function as a system rather than on the level of the single target significantly increases therapeutic potency. In the present study, we detect the target pathway of salvianolic acid B (SalB) *in vivo*. Acute myocardial infarction (AMI) was induced in rats followed by the treatment with 10 mg/kg SalB. Hemodynamic detection and pathological stain, 2-dimensional electrophoresis, MALDI-TOF MS/MS, Western blot, pathway identification, apoptosis assay and transmission electron microscope were used to elucidate the effects and mechanism of SalB on cardioprotection. Higher SalB concentration was found in ischemic area compared to no-ischemic area of heart, correlating with improved heart function and histological structure. Thirty-three proteins regulated by SalB in AMI rats were identified by biochemical analysis and were classified as the components of metabolism and apoptosis networks. SalB protected cardiomyocytes from apoptosis, inhibited poly (ADP-ribose) polymerase-1 pathway, and improved the integrity of mitochondrial and nucleus of heart tissue during AMI. Furthermore, the protective effects of SalB against apoptosis were verified in H9c2 cells. Our results provide evidence that SalB regulates multi-targets involved in the apoptosis pathway during AMI and therefore may be a candidate for novel therapeutics of heart diseases.

## Introduction

Traditionally, the goal in drug development is to design selective compounds that act on single disease target to achieve desired potency with low side effects. This strategy of “one-gene, one-drug, one-disease” turns out to be unsuccessful for the majority of “complex disease” because pathogenesis of many diseases is multi-factorial rather than due to a single cause [1]. Cardiovascular diseases, the leading cause of mortality and morbidity in the world, are one class of such “complex diseases”. Many cardiovascular diseases are the results of relatively subtle dysfunctions of multi physiological pathways [2]. Development of new compounds targeting different pathways and acting as multi-target inhibitors provides new strategies for cardiovascular therapy. Such polypharmacological approach to target cellular function as a whole rather than each individual target within the pathway may increase therapeutic potency [3].

*Salvia miltiorrhiza* has been widely used in clinic in China for the treatment of various microcirculatory disturbance-related diseases, such as cardiovascular disease, cerebrovascular disease, renal dysfunction, liver fibrosis, and diabetic vascular complication [4]. Salvianolic acid B (SalB) is the major water-soluble component extracted from *Salviae miltiorrhizae*. Several lines of *in vitro* experiments have demonstrated biological activities of SalB in promoting cellular proliferation and differentiation, anti-apoptotic, preserving normal cellular functions [5]–[9]. Our recent data showed that SalB protects against cardiac remodeling through inhibition of matrix metalloproteinase-9 and fibrosis [10]. Base on these reports, SalB seems to have pleiotropic effects and may act on multiple molecular targets, making it a suitable candidate for polypharmacology strategy to develop novel cardiovascular therapeutics.

In the present study, we used myocardial infarction as a disease model to evaluate protective effect of SalB on diseased heart. A series of assays were employed to explore the potential mechanism of SalB cardioprotection including biochemical, cardiophysiological, histopathological, and pathway analysis. Our results indicated that SalB regulated multi-targets involved in the apoptosis pathway during acute myocardial infarction and therefore might be a candidate for further polypharmacology research on novel therapeutics of cardiovascular diseases.

## Materials and Methods

### Animal model and SalB treatment

Wistar male rats (230–250 g) were purchased from Shanghai Center of Experimental Animals, Chinese Academy of Sciences. The purity of SalB (purchased from Shanghai Yousi Bio-Tech Co., Ltd.) was more than 99% evaluated by high-performance liquid chromatography (Figure S1) and the chemical structure of SalB was elucidated by ^1^H NMR and ^13^C NMR (Figure S2, S3, S4). AMI was introduced by ligation of the left anterior descending coronary artery near the main pulmonary artery as described previously [11]. Animals were randomly assigned into four groups: sham operated rats given saline (Sham, n = 30), sham operated rats given SalB (Sham-SalB, n = 40), AMI rats given saline (AMI, n = 30), AMI rats given SalB (AMI-SalB, n = 40). Thirty min later and 24 h later after surgery, saline or SalB was administered through intravenous (IV) injection (10 mg/kg). The dose of 10 mg/kg was set according the protection of SalB on ischemic area (Figure S5). Ten out of 40 rats from Sham-SalB or AMI-SalB were used to measure tissue distribution of SalB. This study was carried out in strict accordance with the recommendations in the Guide for the Care and Use of Laboratory Animals of National Institutes of Health. All protocol was approved by Institutional Animal Care and Use Committee at Shanghai Institute of Materia Medica (IACUC number: SIMM-AE-GDA-2010-03).

### Tissue distribution of SalB

Tissue-distribution of SalB in Sham rats or AMI rats was detected. One-hundred-fifty mg of each tissue sample was homogenized in methanol for approximately 1 min (1∶5 ration of tissue to methanol) using a Fluko F6/10 superfine homogenizer. Ultrasonic treatment was applied for 5 min, and then the resulting samples were centrifuged at 3500 rpm for 5 min at 4°C. Supernatant was used to detect SalB concentration by chromatography (Agilent Waldbronn, Germany). An API 4000 triple quadrupole mass spectrometer equipped with a TurboIon Spray ionization interface (Applied Biosystems, Canada) was used for mass analysis and detection. Data acquisition was performed with Analyst 1.4.1 software (Applied Biosystems, USA).

### Measurements of hemodynamic parameters and cardiac output

Twenty-five hours after surgery, the rats were anesthetized, and a Mikro-tipped SPR-320 catheter (Millar Instruments Inc) was inserted through the right carotid artery into left ventricle. Heart rate, mean arterial pressure (MAP), left ventricular systolic pressure (LVSP), end-diastolic pressure (EDP) of rats were recorded by PowerLab 8/30 instrument (ADInstruments, Australia). Maximal rate of pressure development for contraction (+dP/dt_max_) and maximal rate of pressure development for relaxation (−dP/dt_max_) were all calculated from the continuously collected pressure signal. For cardiac output detection, rats were anaesthetized, placed on a heating pad supplied with ventilation. The thymus lobes were pulled apart to expose the aorta. The ascending aorta was dissected and a transonic perivascular MA2.5 PSL flow probe (Transonic Systems, USA) was positioned around the aorta. Appropriate amount of ultrasound transmission gel was injected into the space between the probe and the aorta. Flow signals were then acquired by TS420 flowmeter and PowerLab recording unit.

### Histopathological detection

After detection of hemodynamic parameters, the heart samples were fixed by 4% neutral-buffered paraformaldehyde for 24 h, and the specimens were paraffin-embedded, cut at 5 µm and were stained with haematoxylin and eosin. Photomicrographs were taken using an Olympus BX51 microscope plus Olympus DP71 CCD camera (Olympus Corporation, Japan).

### Two-dimensional electrophoresis

Heart tissues from rat infarct area were pulverized under liquid nitrogen and then detergent soluble proteins were extracted by incubation in lysis buffer. Homogenization was achieved by ultrasonication on ice, then centrifugation at 15, 000×g for 30 min at 4°C. The supernatants were used for two-dimensional electrophoresis as described previously using Bio-Rad 2-DE system [12]. Briefly, 150 µg protein sample was applied for IEF using the ReadyStrip IPG Strips. The strips were placed into a Protein IEF cell and were rehydrated at 50 V for 12 h and then the proteins were separated based on their pI. After IEF, the IPG strips were equilibrated for 15 min in a buffer containing 50 mM Tris-HCL, pH 8.8, 30% glycerol; 7 M urea, 2% SDS and 1% DTT, and then applied on to 12% homogeneous SDS-PAGE gels for electrophoresis using a PROTEIN II xi Cell system (Bio-Rad, USA). The gels were then silver stained using Bio-Rad Silver Stain Plus kit according to the manufacturer's instructions. For each protein sample, three independent electrophoreses were performed to ensure reproducibility.

### Image analysis and MALDI-TOF MS/MS

The silver-stained gels were scanned using a Densitometer GS-800 (Bio-Rad, USA) and the images were analyzed using PD-Quest software (Bio-Rad, USA). Proteins of interest were excised from the gels with EXQuest Spot Cutter (Bio-Rad, USA), and MS analysis was performed as described previously [13]. Briefly, gel pieces were destained for 2 min, washed twice with deionized water and shrunk by dehydration in ACN. The samples were then swollen in a digestion buffer containing 25 mM ammonium bicarbonate and 12.5 ng/µl trypsin at 4°C. After a 30 min incubation, the gels were digested for over 12 h at 37°C. Peptides were extracted twice using 0.1% TFA in 50% ACN, and the extracts were dried under N_2_. For MALDI-TOF MS/MS, peptides were mixed with 0.7 µl MALDI matrix and spotted on the 192-well stainless steel MALDI target plates. MS measurements were carried out on an ABI 4700 Proteomics Analyzer with delayed ion extraction (Applied Biosystems). Data search files were generated and then the database search was performed by using the MASCOT search engine (Matrix Science, United Kingdom). Protein homology identifications of the top hit (first rank) with a relative score exceeding 95% probability and additional hits (second rank or more) with a relative score exceeding 98% probability threshold were retained. Proteins with protein score more than 62 and best ion score more than 30 were accepted.

### Pathway identification

KEGG was used to analysis all differential expressed proteins in 2-DE experiment and a list of target pathways was generated [14]. To explore the biological relevance underlying these target pathways, linked pathways with these target pathways were also retrieved from KEGG database and the pathway networks were constructed using Pajek network visualization software (version 1.25).

### Western blot analysis

Western blot analysis was performed on the infarct samples using antibodies against the following proteins purchased from Cell Signaling: poly(ADP-ribose) polymerase-1 (PARP-1), cleaved PARP-1, IKKα, IKKβ, p-IKKα, NF-κB, GAPDH. Antibody for L-lactate dehydrogenase B (LDHB) was purchased from Abcam and β-actin was from Beyotime. The aliquots of 25 µg protein were electrophoresed on a SDS–PAGE gel and transferred to polyvinylidene difluoride membrane. The membrane was blocked for 1 h in blocking buffer containing 5% non-fat dry milk and 0.07% Tween 20 in Tris-buffered saline (TBS-T), followed by incubation with indicated antibodies in blocking buffer overnight at 4°C. Then, the membrane was washed three times with TBS-T for 30 min and incubated at room temperature for 1 h with secondary antibody. Detection was done using the ECL Western Blotting Detection System (Pierce, USA). The bands density for corresponding protein was quantified using a MiniBis system (DNR Bio-Imaging Systems Ltd, Israel).

### TUNEL assays

To evaluate apoptosis in infarct area of rat heart, terminal deoxynucleotide transferase-mediated dUTP nick-end labeling (TUNEL) assay using FragEL™ DNA Fragmentation Detection Kit was carried out according to the manufacturer's instructions (Calbiochem, USA). Briefly, the heart samples were fixed by incubating in 4% neutral-buffered paraformaldehyde for 24 h, and the specimens were then paraffin-embedded, cut to 5 µm slices. The sections were de-paraffinized, rehydrated, and permeabilized by incubation in solutions containing proteinase K. After equilibration and labeling reaction, the results were evaluated using BX51 fluorescence microscope plus Olympus DP71 CCD camera (Olympus Corporation, Japan). Total cells were visualized at 330–380 nm for DAPI staining, and apoptotic cells were visualized at 465–495 nm for FITC staining.

### Transmission electron microscopy

Hearts were dissected and small pieces from ischemic area were cut and fixed with 2.5% glutaraldehyde in 0.1 M cacodylate buffer for 2 h, post-fixed in 1% osmium tetroxide in 0.1 M cacodylate buffer for 1 h and embedded as monolayers in LX-112 (Ladd Research Industries, USA). Ultrathin sections were cut and contrasted with uranyl acetate followed by lead citrate and observed with a Tecnai 12 BioTwin transmission electron microscope (Philips Electronic Instruments, USA). Random sections were selected for analysis by an electron microscopy technician blinded to the treatments.

### Cell culture and hypoxia challenge

Rat H9c2 cells (Cell bank of Chinese Academy of Sciences, Shanghai) were maintained in Dulbecco's modified Eagle's medium (DMEM) supplemented with 10% fetal calf serum and cultured in 5% CO_2_ at 37°C. All media and culture reagents were products of Gibco (Grand Island, NY) unless specified otherwise. Just before induction of hypoxia challenge, vehicle or SalB was added into the serum free culture media and the cells were placed into a humidified hypoxia incubator (Tri-Gas Incubator, Heal Force) to achieve a low-oxygen environment (95% N2 and 5% CO2) for 24 h. Mitochondrial membrane potential detection and DAPI stain were performed following.

### Mitochondrial membrane potential detection using JC-1

A fluorescent cationic dye, 5,5′, 6,6′-tetrachloro-1,1′, 3,3′-tetraethylbenzimidazolyl-carbocyanine iodide, commonly known as JC-1 (Beyotime), was used to measure mitochondrial membrane potential. After hypoxia treatment, cells grown on cover slips were washed twice with PBS and incubated with 5 µg/ml JC-1 dye in serum-free medium for 20 min at 37°C. Cells were washed three times with PBS and analyzed immediately under a fluorescent microscope after mounting with DAKO mounting medium. JC-1 fluorescence was measured from single excitation (488 nm) with dual emission (shift from green 530 nm to red 590 nm). Green fluorescence reveals the monomeric form of the JC-1 molecule, which appears in the cytosol after mitochondrial membrane depolarization. Red fluorescence is emitted from the aggregation of JC-1 molecules, which is formed in the inner membrane of actively respiring mitochondria. Photomicrographs were taken using an Olympus BX51 microscope plus Olympus DP71 CCD camera (Olympus Corporation, Japan).

### Apoptosis detection by staining of nuclei with DAPI

The nuclei of H9c2 cells were stained with chromatin dye DAPI (Invitrogen, USA). Briefly, after treatment, the cells were fixed with 4% paraformaldehyde for 20 min, washed twice with PBS, and incubated with 10 µg/ml DAPI in PBS at room temperature for 30 min. After three washes, the cells were observed under an Olympus fluorescence microscope (BX51) plus Olympus DP71 CCD camera. Total cells and apoptosis cells per field were counted by a blinded investigator. The quantitative data was expressed as percentage of apoptosis cell per field.

### Statistical analysis

All quantitative data are reported as mean ± S.E. Statistical analyses were performed using one-way ANOVA to compare means, followed by post hoc test. *p*<0.05 was considered to be statistically significant.

## Results

### Accumulation of SalB in the ischemic area of heart

**Figure 1:**
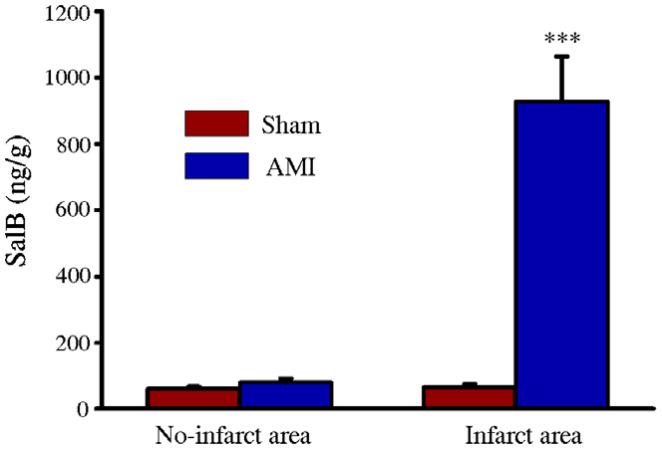
Accumulation of SalB in ischemic area of heart. All the values are expressed as mean ± S.E. ***p<0.001 versus no-infarct area of Sham. n = 10 for every group.

First we analyzed SalB concentration in several major organs. 1 h after intravenous administration of 10 mg/kg SalB, major organs (heart, kidney, and lung) were harvested and tissue homogenate was prepared. The concentration of SalB in heart is 60.8±6.7 ng/g (no-infarct area of Sham); 79.2±12.8 (no-infarct area of AMI); 65.1±9.2 ng/g (infarct area of Sham) and 928.2±135.8 ng/g (infarct area of AMI), respectively. There was no difference of SalB concentration in liver and kidney between Sham and AMI rats, the concentrations were all below 50 ng/g (Figure 1). The higher concentration of SalB at ischemic area of heart promoted us to concentrate this area for further mechanism research.

### SalB improves left ventricle function of AMI rat

**Figure 2:**
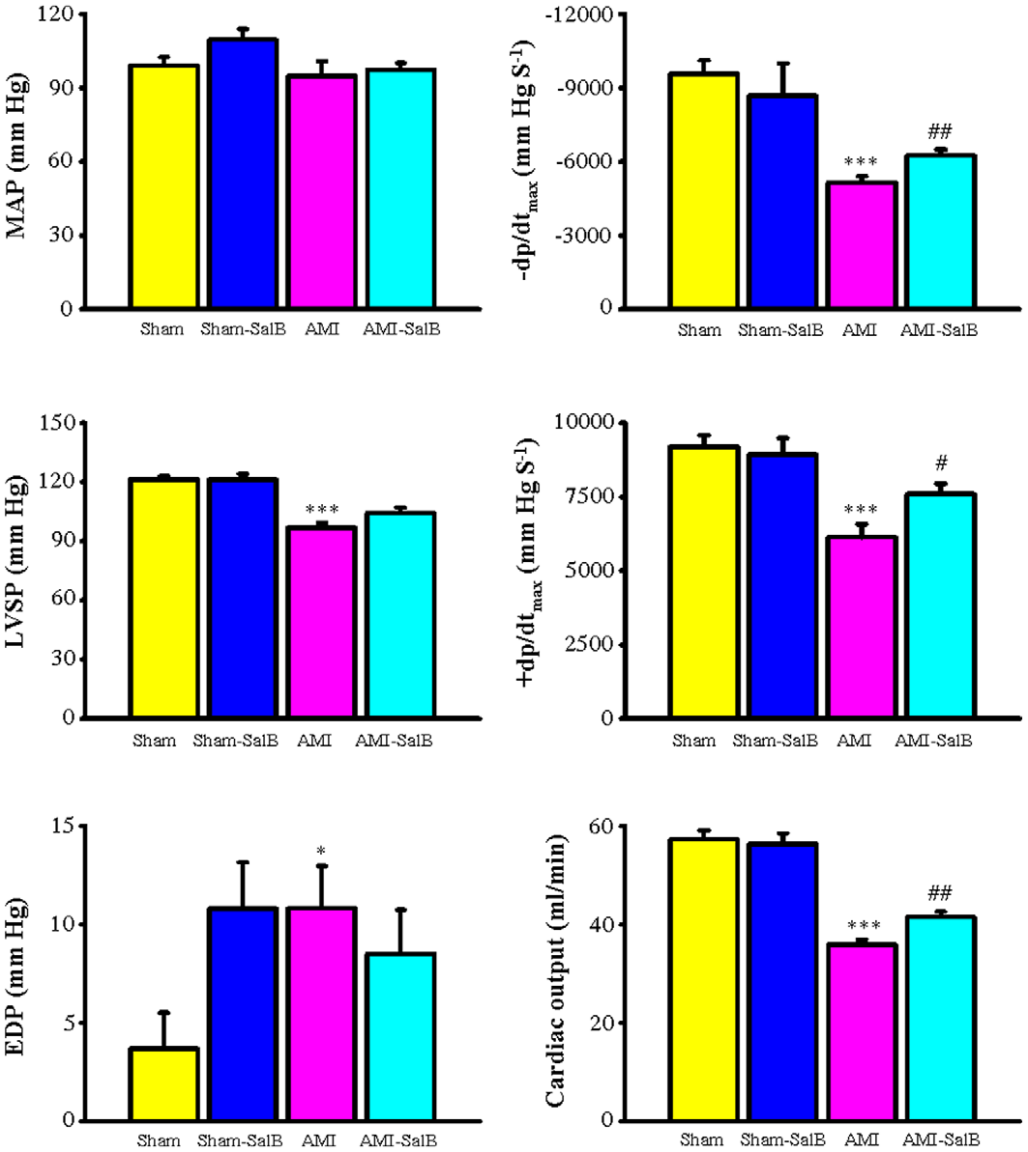
SalB improves cardiac function of AMI rats. Mean arterial pressure (MAP), maximum rate of pressure development for relaxation (−dP/dt_max_), left ventricular systolic pressure (LVSP), maximum rate of pressure development for contraction (+dP/dt_max_), end-diastolic pressure (EDP), and cardiac output were demonstrated. All the values are expressed as mean ± S.E. *p<0.05, ***p<0.001 versus Sham; #*p*<0.05, ##*p*<0.01 versus AMI. n = 30 for every group.

To evaluate the possible protective effect of higher concentration of SalB in heart of AMI rats, we measured several major haemodynamic parameters for left ventricle contractility, including MAP, −dp/dt_max_, LVSP, +dp/dt_max_, and EDP along with cardiac output (Figure 2). Compared to rats in the Sham group, left ventricle dysfunction in AMI rats was observed with significant decrease of −dp/dt_max_ (−5153.2±257.4 mmHgS^−1^ versus −9578.0±546.0 mmHgS^−1^, *p*<0.001), LVSP (96.7±2.5 mm Hg versus 121.4±1.8 mm Hg, *p*<0.001), +dp/dt_max_ (6134.1±441.6 mmHgS^−1^ versus 9184.6±393.9 mmHgS^−1^, *p*<0.001), and increase of EDP (10.8±2.1 mmHg versus 3.7±1.8 mmHg, *p*<0.05). SalB treatment reduced the degree of impairment of left ventricle function with the elevation of values of −dp/dt_max_ (−6260.2±233.3 mmHgS^−1^ versus −5153.2±257.4 mmHgS^−1^, *p*<0.01), +dp/dt_max_ (7603.2±348.1 mmHgS^−1^ versus 6134.1±441.6 mmHgS^−1^, *p*<0.05), compared with the AMI group. No detectable difference in MAP was observed among all groups. No significant difference in hemodynamic parameters was found between the Sham-SalB group and the Sham group. To further evaluate cardiac function of rats from different treatment groups, transonic flowmeter was used to measure the cardiac output. The value of cardiac output for AMI rats decreased to 35.9±1.0 ml/min from 57.2±1.9 ml/min observed in rats from the Sham group (*p*<0.001). With the SalB treatment, the cardiac output in these rats improved to 41.6±1.0 ml/min (*p*<0.01).

### SalB improves left ventricle structure of AMI rat

**Figure 3:**
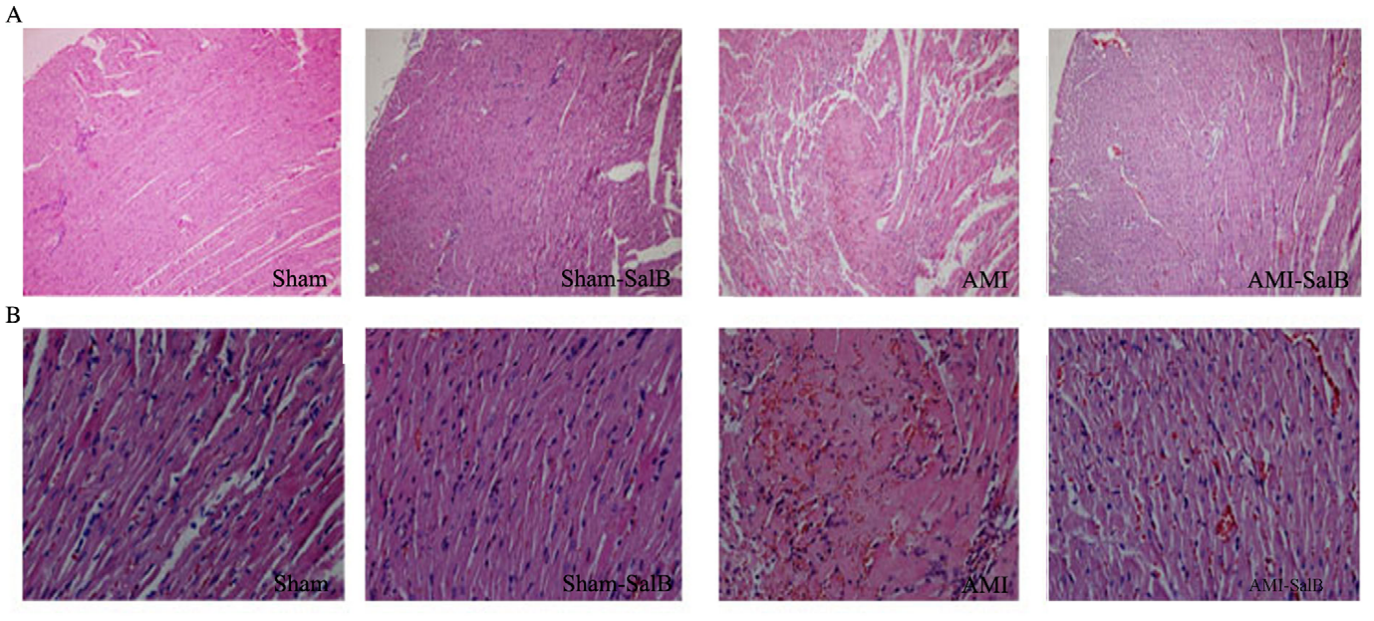
SalB improves heart structure of AMI rat detected by H&E stain. Representative photomicrographs from heart tissue of Sham, Sham-SalB, AMI, AMI-SalB groups were shown. (**A**) 100× magnification. (**B**) 400× magnification. n = 10 for each group.

To explore the structural basis leading to observed impairment of left ventricle function, histopathological evaluation of heart samples from all groups was performed. The cell alignment was regular and no obvious damage was detected in the myocardium of Sham rats and Sham-SalB rats. However, marked cellular degeneration, interstitial edema and coagulation necrosis in the ischemic region were observed in AMI rats. Upon SalB treatment, there was a profound increase of cross striation and cell integrity in samples from AMI-SalB rats, suggesting that SalB markedly ameliorated the myocardial damage induced by AMI (Figure 3).

### Differential protein expression profile among different groups

**Figure 4:**
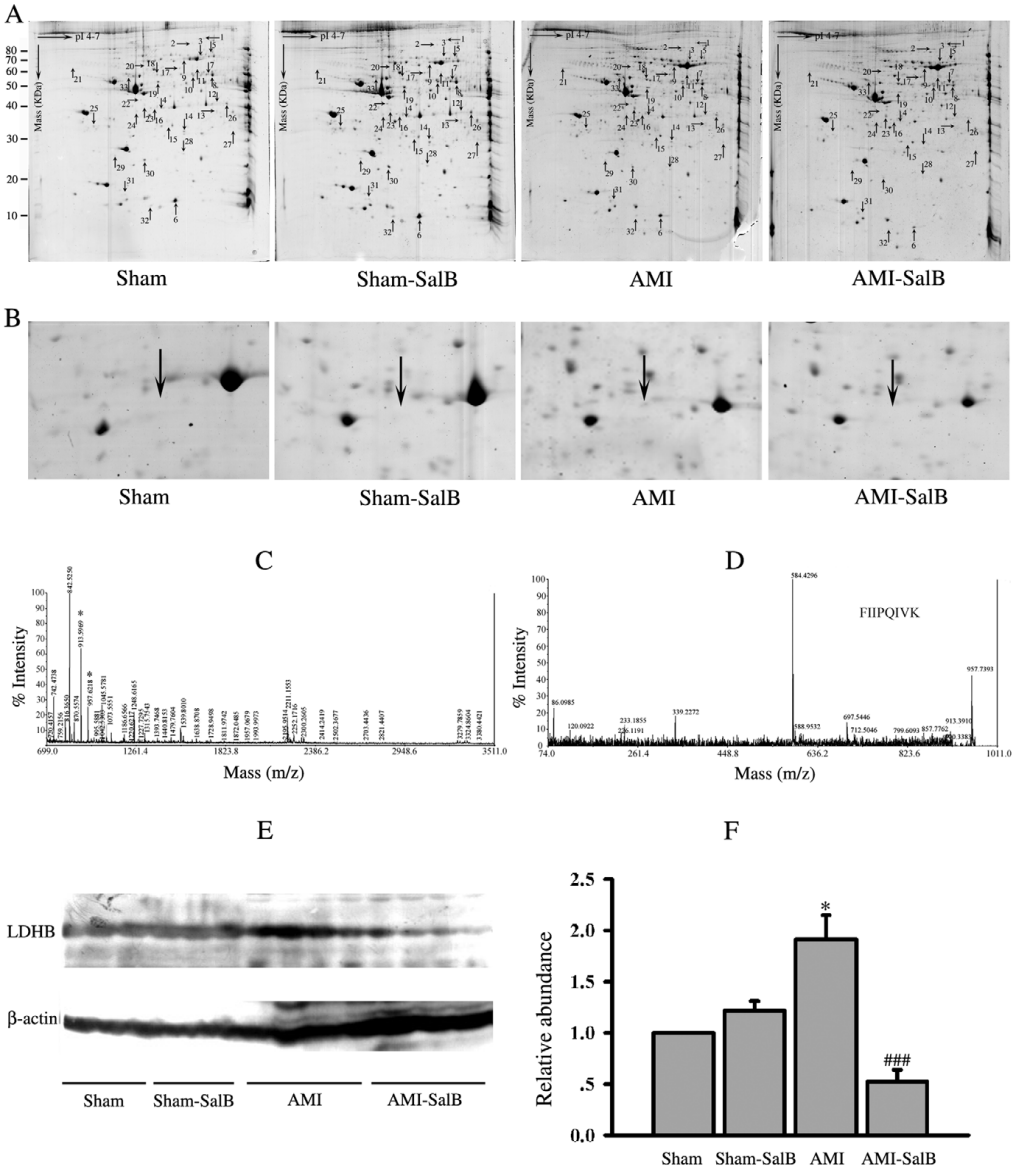
Differentially expressed proteins resolved by 2-DE analysis, identified by MS and Western blot. (**A**) Representative 2-DE gel images from Sham, Sham-SalB, AMI and AMI-SalB. Differentially expressed spots chosen for further MALDI-TOF MS/MS analysis were indicated by arrows. n = 6 for every group. (B) Close-up image of protein spot 4 on different treatments. (C) MALDI-TOF MS/MS spectra of the spot 4 cut from the 2-DE gel resulted in the identification of LDHB. (D) MS/MS profile of the peptide with a mass of 957.6 Da from spot 4. (E) Western blot of LDHB in different treatments, one lane represents one rat. n = 10 for every group. (F) Quantitative data of LDHB for Western blot. *p<0.05 versus Sham; ###*p*<0.01 versus AMI. n = 10 for each group. At least three time experiments were repeated and the representative figures were shown.

Left ventricular myocardial samples taken from ischemic area were subjected to three independent 2-DE analyses under the same conditions to assure reproducibility. More than 560 protein spots were detected in samples of each rats from each treatment group (n = 5). Between samples from Sham and AMI rats, thirty-three protein spots exhibited significantly difference in intensity (*p*<0.05). Among those, sixteen of the thirty-three spots were considerably up-regulated while other seventeen spots were significantly down-regulated in samples from AMI rats compared to those from Sham rats (Table 1). These spots were labeled and further analyzed using MALDI-TOF MS/MS (Figure 4A; Table 2). The close-up image for spot 4 was shown and the protein species for this spot was identified as LDHB in the experiment described below (Figure 4B).

**Table 1:** Statistical analysis of 2-DE purified proteins changing in abundance following 24 h MI.

Spot no.	PPM			
	Sham	Sham-SalB	AMI	AMI-SalB
1	0.031±0.060	0.047±0.080	77.44±20.41**	43.53±31.79#
2	0.031±0.060	0.047±0.080	129.23±51.58**	64.88±37.44#
3	65.95±34.70	108.58±57.93	178.07±64.22**	375.03±222.66#
4	0.017±0.039	1.62±4.28	21.97±30.42*	0.04±0.052#
5	60.05±19.96	81.72±61.24	95.14±20.92**	59.20±29.08##
6	10195.07±4565.27	11004.51±4404.93	4084.71±2940.35**	1999.19±585.55#
7	0.031±0.060	297.37±220.76**	552.39±297.49**	242.35±77.83#
8	5123.79±2121.37	5493.86±2338.04	3523.49±1814.43*	1925.07±575.30##
9	608.76±815.45	1061.71±1353.48	2982.86±2114.23**	962.09±595.63#
10	1511.33±607.93	792.08±79.81**	687.97±409.98**	414.32±170.65#
11	106.34±76.43	154.80±107.02	210.64±121.70*	103.03±33.22#
12	0.015±0.04	0.040±0.08	54.89±29.99**	0.054±0.07##
13	129.39±54.38	135.09±86.40	68.23±28.87**	37.35±31.95#
14	781.38±250.70	561.72±180.01**	292.16±170.23**	139.26±41.86##
15	42.92±34.02	33.32±23.70	0.093±0.08**	17.19±10.94**
16	53.20±29.99	48.99±29.58	104.55±47.66**	64.22±30.20#
17	241.55±115.54	269.82±123.72	446.34±199.24**	323.65±130.49
18	391.19±237.48	346.71±211.54	1057.86±403.52**	1451.14±435.50#
19	1687.69±845.21	1428.55±845.21	310.11±149.48**	189.72±67.31#
20	73.58±36.75	24.07±65.91*	159.28±75.45**	247.92±94.95#
21	83.01±67.88	118.02±90.42	296.84±244.00**	166.41±158.52
22	245.95±110.24	180.27±104.29	98.27±44.93**	50.50±16.83##
23	113.45±71.38	113.25±71.12	499.88±221.35**	834.65±241.87##
24	152.40±77.40	73.01±83.24**	87.38±50.26*	37.85±23.21##
25	407.24±257.81	447.06±306.43	210.85±66.85**	129.36±65.89##
26	1443.74±522.57	1175.32±553.83	311.30±141.47**	173.95±59.41##
27	60.58±56.25	61.02±53.62	105.84±55.73*	39.78±21.18##
28	218.34±96.84	162.44±79.81	139.34±52.49*	98.96±25.96#
29	186.22±73.60	147.78±65.38	84.43±26.32**	54.68±34.47#
30	1407.17±653.52	1545.66±773.55	393.71±255.81**	222.77±98.22#
31	77.76±45.55	68.01±39.41	33.33±17.79**	15.12±10.97##
32	341.83±200.10	260.62±129.44	77.42±71.56**	23.63±22.87#
33	303.74±182.15	555.25±633.07	172.83±104.76*	63.01±35.91##

*p<0.05,

**p<0.01,

***p<0.001 versus Sham;

#*p*<0.05,

##*p*<0.01,

###*p*<0.001 versus AMI.

**Table 2:** Identification of proteins significantly altered in ischemic area of heart after 24 h MI.

Spot No.	Identification	Accession No. of NCBI database	Theoretical MW (Da)	Theoretical p*I*	protein score
1	vinculin (predicted), isoform CRA_a	149031250	123555.8	5.54	99
2	actinin alpha 2	157951643	103768.3	5.31	104
3	mitochondrial ribosomal protein L45	157786676	35417.4	9.44	64
4	L-lactate dehydrogenase B	6981146	36589.1	5.7	112
5	TNF receptor-associated protein 1 precursor	84781723	80410.8	6.56	219
6	fatty acid binding protein 3, muscle and heart	13162363	14765.6	5.9	414
7	mitochondrial aldehyde dehydrogenase precursor	45737868	55565.2	7.63	246
8	Malate dehydrogenase 1, NAD (soluble)	37590235	36461	5.93	116
9	Serum albumin	124028612	68686.1	6.09	297
10	enolase 1, (alpha) isoform 1	56107	47086.2	6.16	150
11	similar to T-complex protein 1 subunit alpha (TCP-1-alpha) (CCT-alpha)	109472340	51646.1	7.98	239
12	enolase 1, (alpha) isoform 1	158186649	47098.2	6.16	301
13	glycerol-3-phosphate dehydrogenase 1 (soluble)	57527919	37428.2	6.16	112
14	peroxiredoxin 6	16758348	24803	5.64	343
15	NADH dehydrogenase (ubiquinone) Fe-S protein 3	157817227	30207.6	7.07	134
16	rCG51928, isoform CRA_b	149030414	27055.4	5.37	104
17	protein disulfide isomerase associated 3, isoform CRA_a	149023097	53553	7.1	356
18	vitamin D-binding protein precursor	51260133	53483	5.65	145
19	creatine kinase, brain, isoform CRA_a	149044074	35862.1	6.27	274
20	Death associated protein 3	55249775	44466	9.08	60
21	calreticulin, isoform CRA_b	149037838	24484.2	5.38	92
22	Temporarily Assigned Gene name family member (tag-58)	109492050	574015.9	5.11	70
23	Ba1-647	33086640	42447.5	6.11	85
24	pyrophosphatase (mapped), isoform CRA_b	149038730	34007	5.21	91
25	tyrosine 3-monooxygenase/tryptophan 5-monooxygenase activation protein, gamma polypeptide	9507245	28284.9	4.8	154
26	aldo-keto reductase family 1, member B1	6978491	35774.3	6.26	203
27	rCG23609, isoform CRA_b (Myosin)	149063939	216384.4	5.68	65
28	adenine phosphoribosyltransferase	61556832	19533.4	6.17	246
29	glyoxalase 1	46485429	20806.3	5.12	121
30	Chain A, Rat Phosphatidylethanolamine-Binding Protein	158428857	21060.5	5.47	207
31	RAN binding protein 2	109509431	344179.5	5.73	65
32	fatty acid binding protein 3, muscle and heart	13162363	14765.6	5.9	303
33	Desmin	38197676	53390.1	5.21	539

During MI, damaged myocardial cells release LDHB into the bloodstream and causes LDHB level to increase. LDHB has fundamental importance in the regulation of heart metabolism and further involves in the regulation of heart function [15]. Since LDHB is generally used as an index for myocardial infarction, we chose LDHB as the candidate protein to verify the data of 2-DE gels. The representative MALDI-TOF MS/MS spectrum for LDHB protein retrieved from the 2-DE gel (Figure 4C) and the detail MS/MS profile of the peptide with a molecular mass of 957.6 Da (Figure 4D) were shown. In addition, Western blot analysis was also used to confirm 2-DE results, complementary to MALDI-TOF MS/MS. Representative blots (Figure 4E) and quantitative result (Figure 4F) of Western blot analysis on LDHB protein were shown. Overall, 1.91±0.58 fold increase in LDHB expression was found in AMI rats compared with Sham rats (*p*<0.05), and SalB treatment reduced the level of LDHB expression compared with AMI (*p*<0.001), suggesting the same expression pattern of LDHB detected by Western blot and 2-DE gel (Table 1).

### SalB protects AMI heart through regulation on metabolism and apoptosis pathways

**Figure 5:**
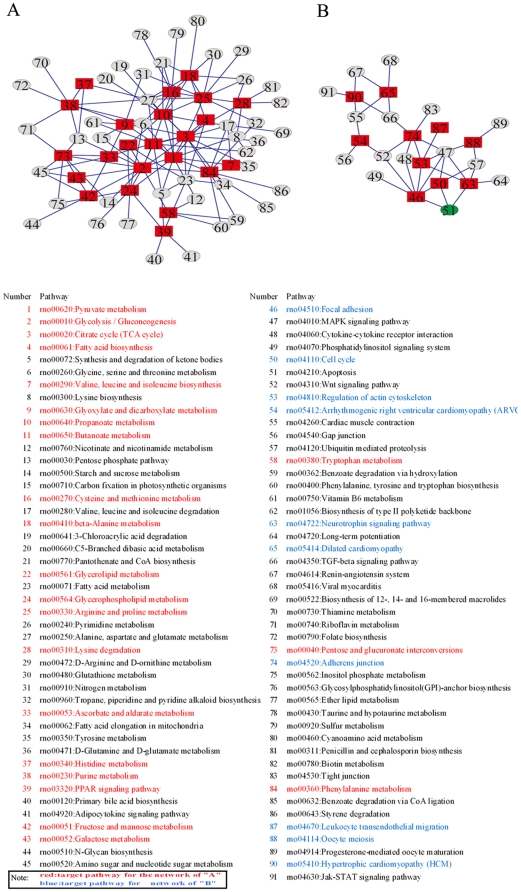
Bioinformatic analysis of proteins identified on 2-DE from the hearts of rats with different treatment. (A) Metabolism pathway network was constructed with 23 target pathways. (B) Apoptosis pathway network was constructed with 11 target pathways. Red rectangles represent target pathways; grey ellipses represent conjunctive pathways. The apoptosis pathway (number 51) was emphasized in green ellipse.

Based on protein expression profile illustrated in 2-DE analysis, we deduced target pathways based on proteins listed in Table 1 and constructed two main pathway networks related to metabolism and apoptosis according KEGG and Pajek analysis. In which the conjunctive pathways were constructed to link the target pathways. The metabolism network (Figure 5A) included 23 target pathways marked by red rectangle and other conjunctive pathways marked by grey ellipse; while the apoptosis network (Figure 5B) included 11 target pathways marked by red rectangle and other conjunctive pathways marked by grey ellipse. The detail information of each pathway was listed underneath, metabolism related pathways in red and apoptosis related in blue. It is very prominent that 3 target pathways (number 46, number 50, number 63) were found to directly connect with apoptosis pathway (number 51; green ellipse), suggesting that anti-apoptosis mechanism may play a key role in SalB cardioprotection.

### SalB protects cardiomyocytes from apoptosis

**Figure 6:**
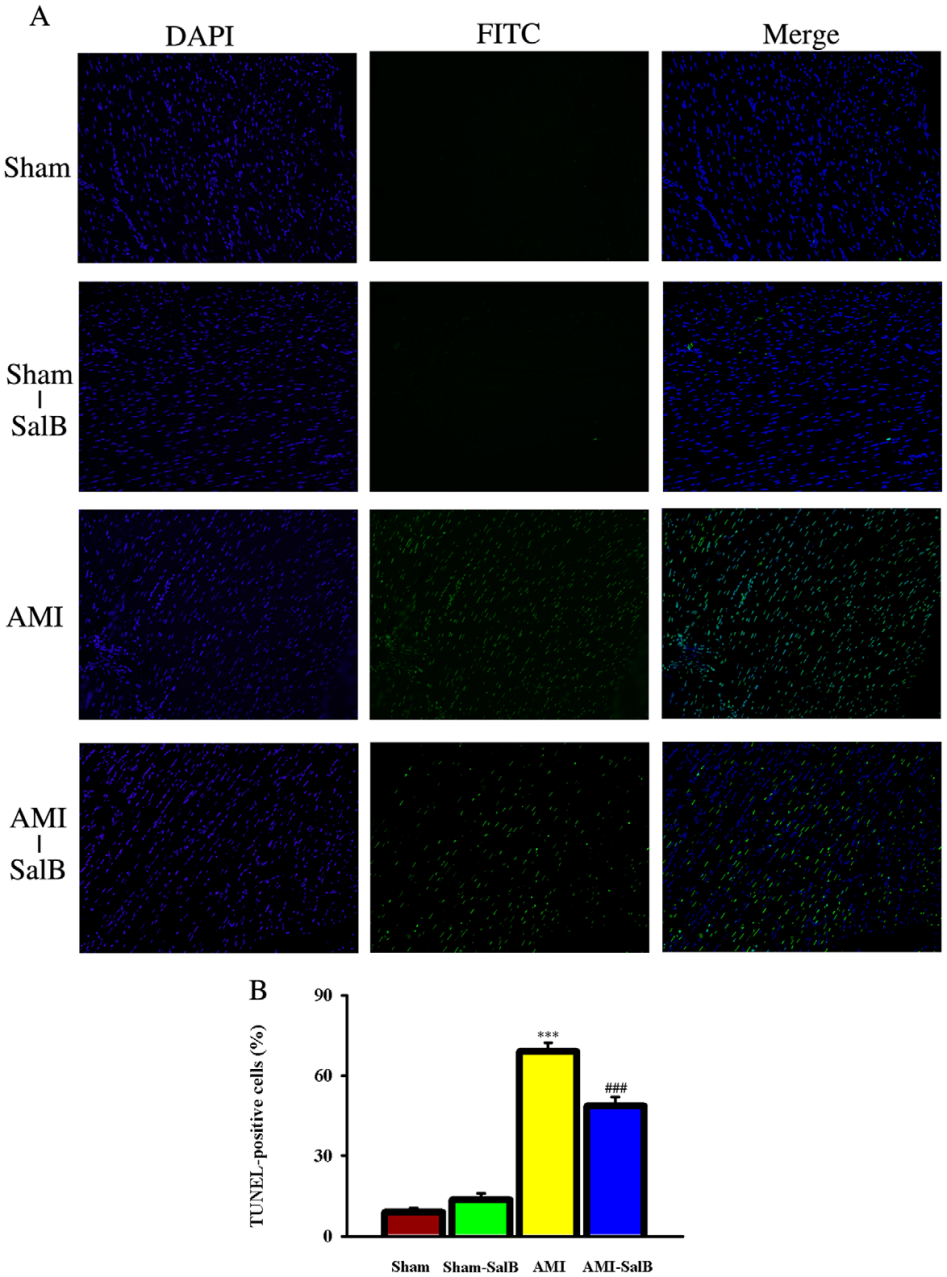
SalB protected cardiomyocytes from apoptosis detected by TUNEL assay in ischemic area of heart. (A) Representative pictures of each treatments. The total cells were identified by DAPI stain, and the positive apoptosis cells were identified by FITC stain. (B) Quantitative data of apoptosis cells. ***p<0.001 compared with Sham, ###*p*<0.001 compared with AMI. n = 10 for each group. At least three time experiments were repeated and the representative figures were shown.

To further investigate the potential anti-apoptosis mechanism for cardio-protection of SalB, we employed *in situ* TUNEL assay to examine apoptosis cardiomyocytes from different treatment groups (Figure 6A). Higher percentages of apoptotic cells were found in AMI rats (69.0±3.3%) compared with Sham rats (8.9±1.5%) (*p*<0.001). SalB treatment reduced the percentage of apoptotic cells to 48.7±3.2%, (*p*<0.001) comparing to AMI (Figure 6B). These data provided the direct evidence that cardio protection of SalB was related to its inhibition on apoptosis.

### Regulation of SalB on apoptosis proteins in ischemic area of heart

**Figure 7:**
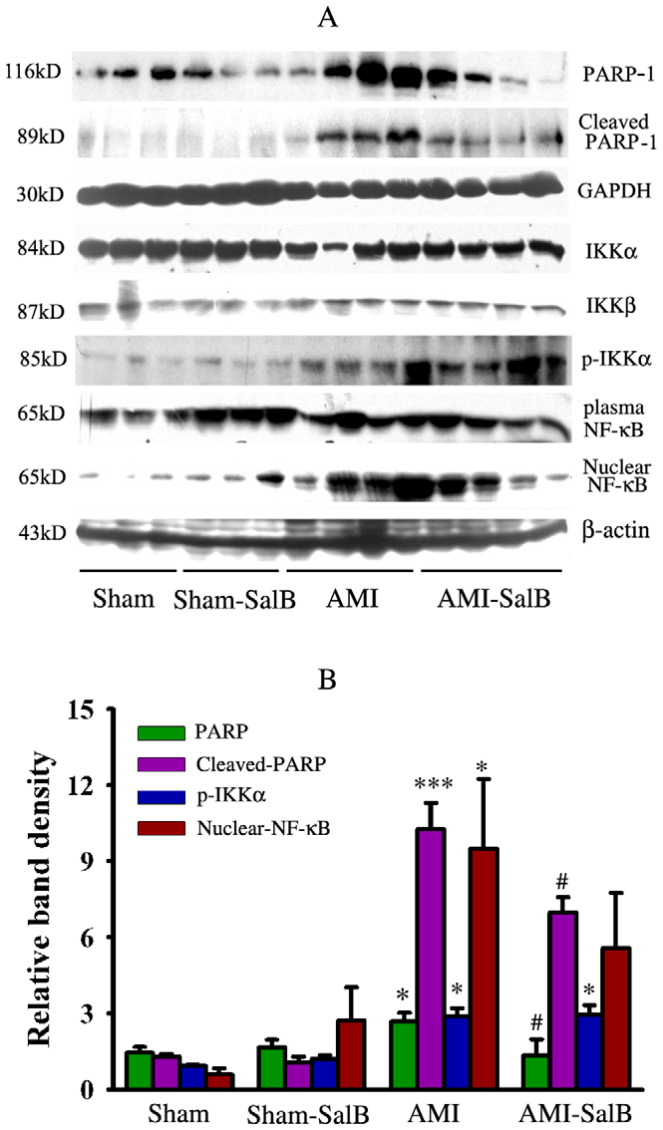
Regulation of SalB on the protein expression of PARP-1 an NF-kB signal pathway. (A) Representative micrograph of Western blot for PARP-1, cleaved-PARP-1, IKKα, IKKβ, p-IKKα, plasma NF-κB, nuclear NF-κB. (B) Quantitative data of Western blot for relative proteins. *p<0.05, ***p<0.001 compared with Sham, #*p*<0.05 compared with AMI. n = 10 for each group. At least three time experiments were repeated and the representative figures were shown.

We further examine the effects of SalB on PARP-1 and NF-κB, two pivotal signal pathways for apoptosis. Protein samples from infarct area of heart were subjected to SDS-PAGE and Western blot analysis. For proteins in the PARP-1 signal pathways, significant elevation in expression level of PARP-1 (*p*<0.05) and cleaved-PARP-1 (*p*<0.001) was found in samples from AMI rats compared to that from Sham rats; SalB treatment led to a significant decrease in expression level of PARP-1 (*p*<0.05) and cleaved-PARP-1 (*p*<0.001) compared to the AMI group (Figure 7A and 7B). For NF-κB signal pathway, significant elevation in levels of p-IKKα (*p*<0.05) and nuclear NF-κB (*p*<0.05) was found in samples from AMI rats compared to that from Sham rats, while no significant change was found between the AMI and AMI-SalB groups (Figure 7A and 7B). For IKKα, IKKβ and plasma NF-κB, no significant difference was found between different treatment groups.

### SalB protects the integrity of mitochondria, nucleus and intercalated disk of heart tissue

**Figure 8:**
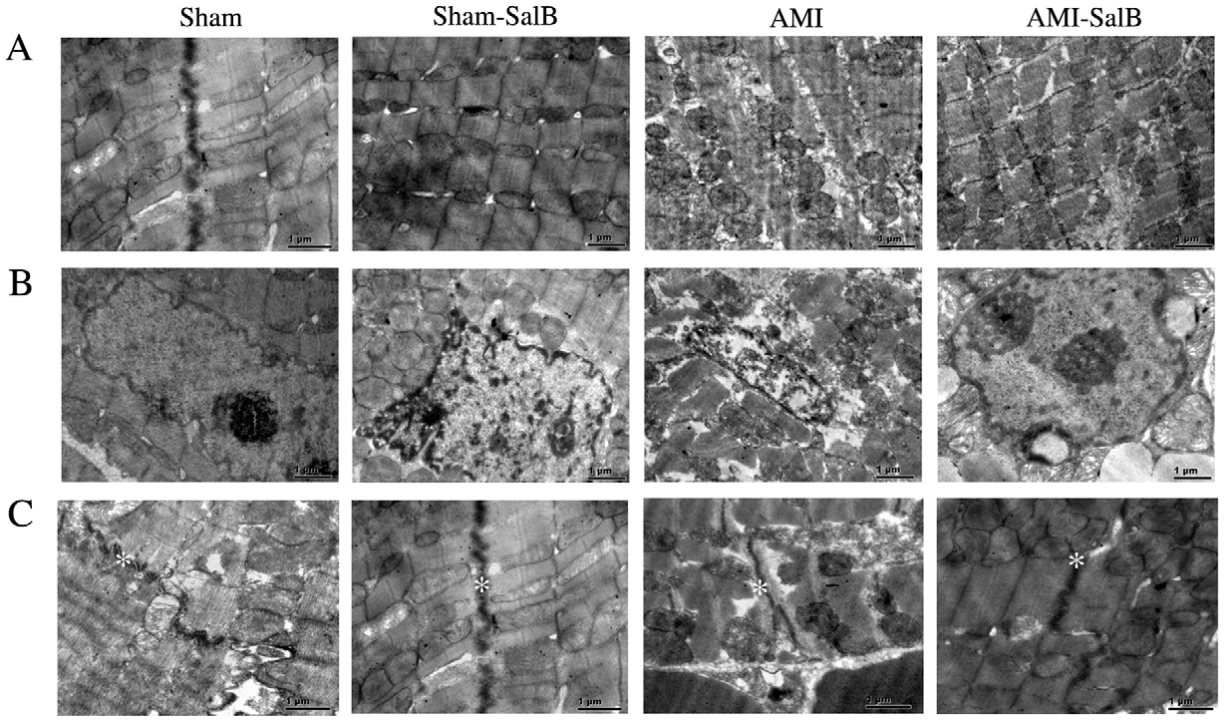
SalB improved ultra-structure of ischemic area of heart. (A) Electron micrograph of sarcomere and mitochondria. (B) Electron micrograph of nuclei. (C) Electron micrograph of intercalated disks marked by asterisk. n = 6 for each group. The representative figures were shown.

Because mitochondria and nucleus are two main intracellular organelles related to apoptosis, it becomes interesting to us to explore the potential protective effect of SalB on the integrity of mitochondria and nucleus during AMI. The structures of mitochondria and nucleus were examined using transmission electron microscopy. The sarcomeres and mitochondria were in regular orientation and homogenous length in Sham and Sham-SalB rats. However in AMI rats, mitochondrial matrix of cardiomyocytes showed severe damage. The mitochondrial cristae showed partial disruption with otherwise normal morphology in AMI-SalB rats (Figure 8A). Nuclei structure appeared to be normal in Sham and Sham-SalB rats with a normal nuclear size, but nuclei from AMI rats displayed an irregular, folded nuclear envelope. An improved nuclei morphology and homogenous nuclei matrix were observed in AMI-SalB rats (Figure 8B). Intercalated disks are highly organized cell-cell structures, which connect cardiomyocyte to one another. A long and integral intercalated disk with many microprojections was observed in Sham and Sham-SalB rats. A convoluted and dissociated intercalated disk with few microprojections was observed in AMI rats, while, SalB treatment improved the structure of intercalated disk (Figure 8C).

### SalB protects mitochondria function and against apoptosis in H9c2 cells

**Figure 9:**
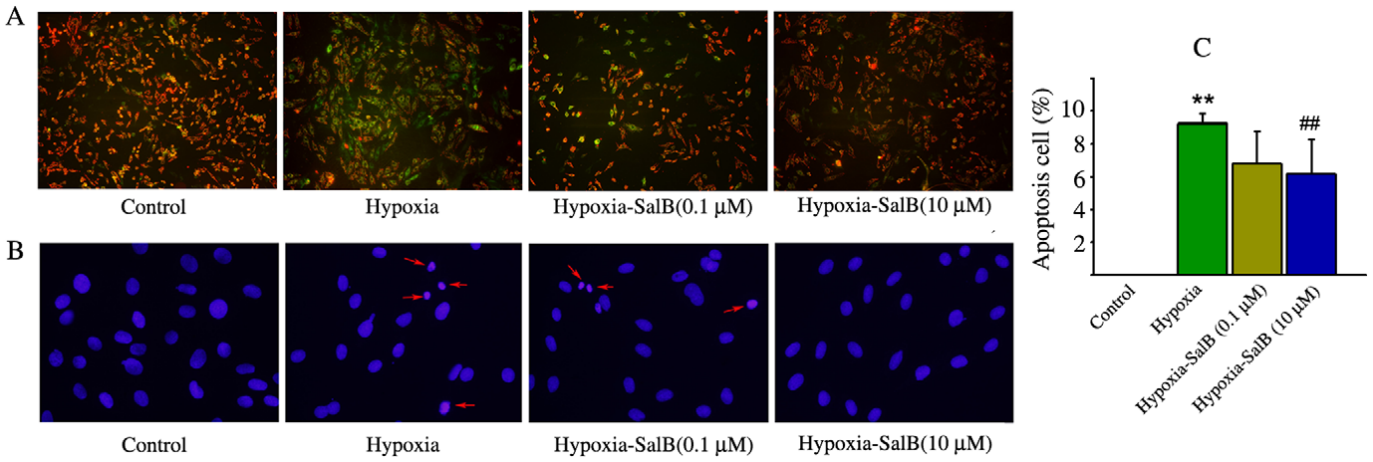
SalB treatment protected mitochondria function and against apoptosis. (A) Detection of mitochondria membrane potential using JC-1. (B) Detection of apoptosis using DAPI. (C) Quantitative data of apoptosis cells. **p<0.01 compared with Control, ##*p*<0.01 compared with Hypoxia. At least three time experiments were repeated and the representative figures were shown.

To further confirm the protection of SalB on cardiac cells, H9c2 cells were used. The change in mitochondrial membrane potential was measured using JC-1 that exists as aggregates in mitochondria with normal potential resulting in red fluorescence or as cytoplasmic monomers at low mitochondrial potentials resulting in green fluorescence. Mitochnodrial Mitochondrial dysfunction occurs at the beginning of the death signaling pathway. Normoxic control cells exhibited numerous brightly stained mitochondria that emitted red/orange fluorescence. With hypoxia treatment, clear formation of monomeric JC-1 was observed, which is indicative of loss of membrane potential. SalB treatment attenuated this hypoxia induced formation of JC-1 monomers (Figure 9A), suggesting the protective effects of SalB on mitochondrial function. DAPI staining was further used to detect the influence of hypoxia on the nuclear changes of H9c2 cells. As shown in Figure 9B, nuclei were in the normal phenotype demonstrating bright and homogenous fluorescence at normoxic culture condition. The nuclei fluorescence of apoptosis cell became bright, fragmented and condensed. The quantitative data indicated that the apoptotic nuclei were increased after 24 h hypoxia treatment, while this process was inhibited by SalB (Fig. 9C).

## Discussion

There have been a number of reports from *in vitro* studies illustrating benefit of SalB treatment in different disease and evidence points to the possibility that SalB may act on multiple targets leading to a concerted positive effect. However, the particular pathway and underlying mechanism has yet to be elucidated. In this study, we attempted to identify potential target pathways of SalB in cardioprotection *in vivo*.

Using the AMI rat model we employed the study and observed a significant improvement, upon SalB treatment, in histopathology, cellular morphology, as well as cardio function of disease heart in AMI rats, along with higher concentration of SalB in heart tissues. As alternation in histology and function of AMI heart can be expected to be accompanied with considerable change in protein expression profile, we then set to conduct proteomics analysis on samples from infarct area of heart. Thirty-three protein species (out of a total of over 560 protein species on 2-DE gels) met the criteria of 1) have a significantly different expression level between Sham and AMI rats, and 2) have a significantly different expression level between AMI and AMI-SalB rats. After characterizing each of these protein species, we constructed two main pathway networks using KEGG and Pajek analysis tools. The 33 proteins identified were found to be largely involved in energy metabolism and apoptosis, suggesting that disturbance of these two pathways may be representative during AMI, as well as SalB treatment. Further analysis on pathway networks revealed that three target pathways were directly linked to apoptosis, suggesting that apoptosis may be the major pathway for cardioprotection of SalB.

We then focused on SalB effect on two main apoptosis pathways. In AMI rats, proteins including KKα, KKβ, NF-κB, p-IKKα in the NF-κB pathway were not significantly affected by SalB. Between AMI and AMI-SalB rats, however, there were significant differences in expression levels of PARP-1 and clearved-PARP-1 proteins in the PARP-1 pathway, implying the involvement of PARP-1 signal in SalB cardio-protection. PARP-1 is an abundant nuclear protein best known to facilitate DNA base excision repair [16]. Under pathophysiological conditions, over-activation of PARP-1 results in unregulated poly (ADPribose) synthesis and widespread cell death [17]. In addition, increased activation of PARP-1 has been implicated in the pathogenesis of acute and chronic myocardial dysfunction [18]. PARP-1 inhibitor has been demonstrated to more effectively decrease the myocardial remodeling than enalapril treatment [19]. Our findings from this study were consistent with previous reports that higher PARP-1 expression level correlated with disease progression while the decrease in PARP-1 expression in SalB treated AMI rats was evident along with cardio-protection. The specific target, and in-depth mechanism of SalB down-regulation on PARP-1 need to be further explored.

The tissue distribution of SalB was investigated for the first time in animal with myocardial infarction. Normal rats have been used to detect SalB tissue distribution after oral administration of SalB (300 mg/kg), and no detectable SalB was found in heart [20]. The permeability of SalB is limited in intestinal so its oral bioavailability is only 3.90% in rats [21]. Therefore, SalB concentrations in blood were similar by oral administration (300 mg/kg) [20] and intravenous injection (10 mg/kg) in our present study. In the present study, we compared the distribution of SalB in heart between normal rats and AMI rat and found very high accumulation of SalB in ischemic area compared with the other area of heart. Myocardial ischemia can cause a lot of pathophysiological changes, such as the enhanced permeability of the cell membrane, the decreased pH value within the ischemic zone. It is known that the stability of SalB increases with pH value decrease. It is likely that changed cell membrane permeability enables SalB penetrate into the ischemic area and decreased pH value enable SalB stay at ischemic area. Even though we did not explore the mechanism why so difference on SalB tissue distribution between normal rat and AMI rat at present stage, it is also an interesting question for further research.

Data from our previous studies demonstrate that SalB improves heart contractility and decreases heart fibrosis in rats with heart failure [10]. Since heart failure is the end stage of heart disease, it is of great importance to known the effects and mechanism of SalB on cardio-protection at earlier stages of heart diseases. The present study is the first to use proteomics technology to conduct mechanistic research on cardioprotection of SalB in animal model. We provided direct evidence supporting our conclusion that the protective effects of SalB are related with its inhibition on apoptosis. Taken together, our results suggest that SalB is a promising lead compound for polypharmacology research on cardiovascular therapy.

## Supporting Information

Figure S1 **The representative chromatogram of high-performance liquid chromatography for SalB.**
(TIF)

Figure S2 **^1^H NMR spectrum of SalB.**
(TIF)

Figure S3 **^13^C NMR spectrum of SalB.**
(TIF)

Figure S4 **Structure elucidation of SalB.** (A) Chemical structure of SalB. (B) ^1^H NMR (400 MHz) and ^13^C NMR (100 MHz) spectral data for SalB.
(TIF)

Figure S5 **SalB decreased infarct sizes dose dependently.** (A) Representative photographs of triphenyltetrazolium chloride stained rat heart after AMI injury. (B) Graphic representation of left ventricle infarct size expressed as percentage of total ischemic area in each group (n = 10). # p<0.05 vs. AMI rats, *** p<0.001 vs. Sham rats.
(TIF)
