# Supplementary figures and images for: Cardio-Protection of Salvianolic Acid B through Inhibition of Apoptosis Network
Source: PLoS One. 2011 Sep 6;6(9):e24036. doi: 10.1371/journal.pone.0024036 (PMC3167815; doi:10.1371/journal.pone.0024036)

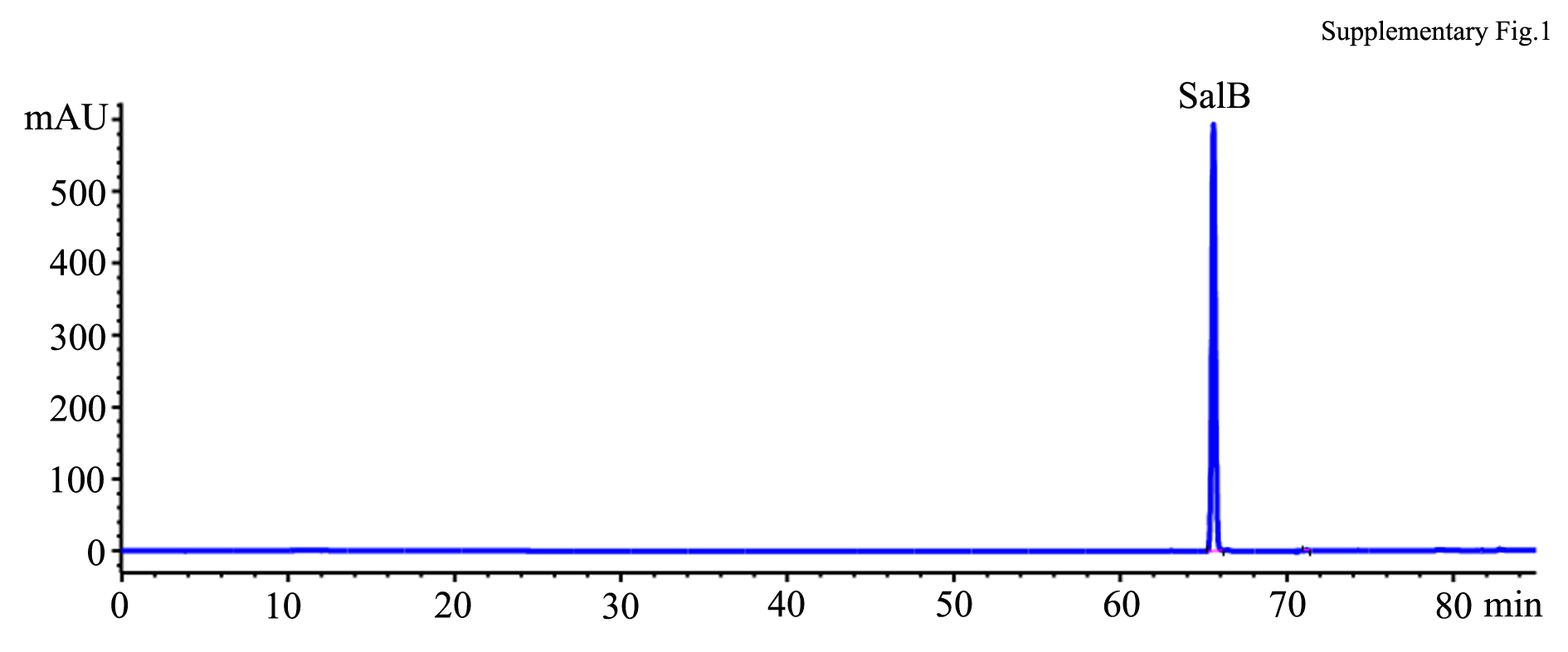

Supplement: Figure S1 — The representative chromatogram of high-performance liquid chromatography for SalB. (TIF) [file pone.0024036.s001.tif]

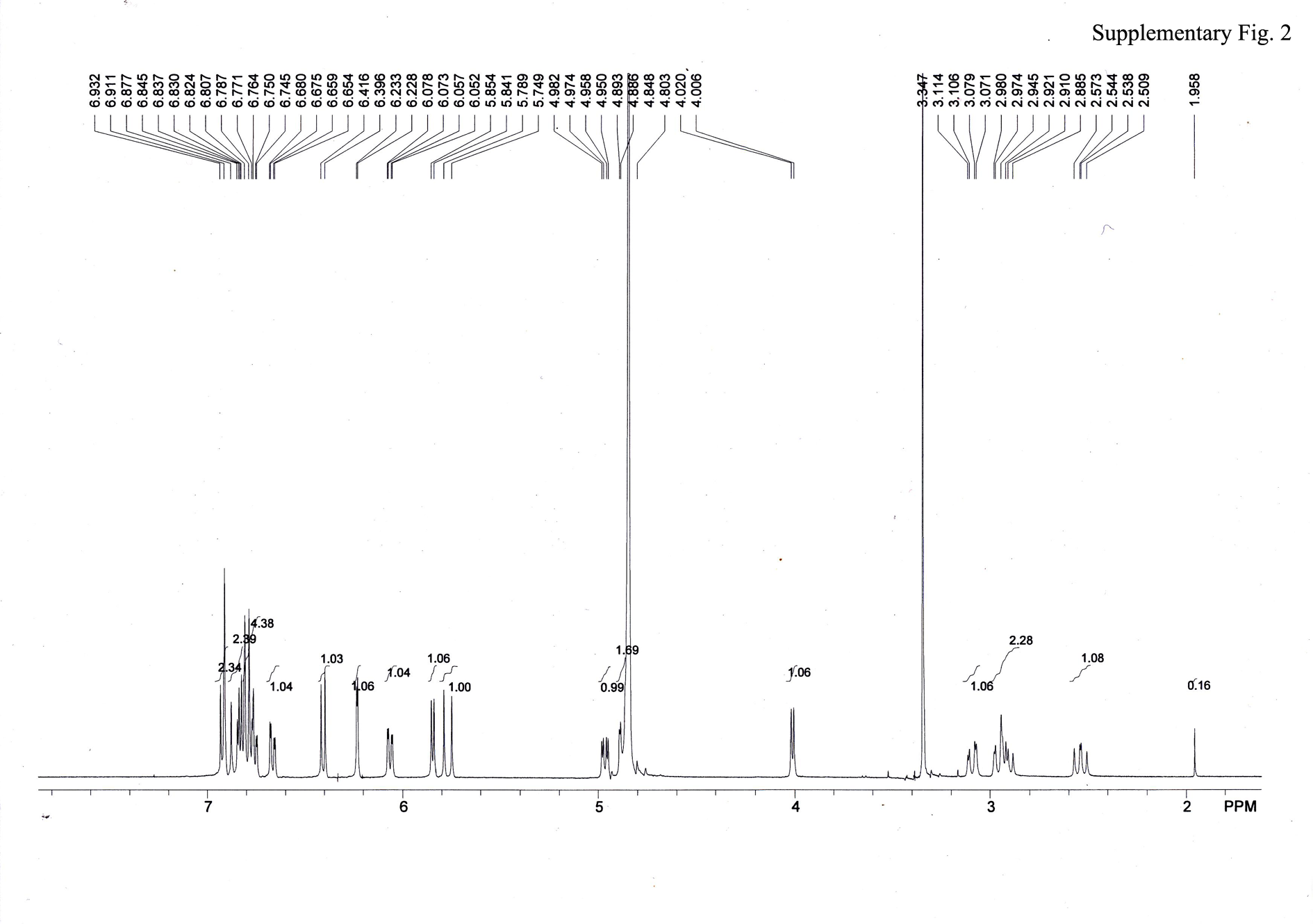

Supplement: Figure S2 — 1H NMR spectrum of SalB. (TIF) [file pone.0024036.s002.tif]

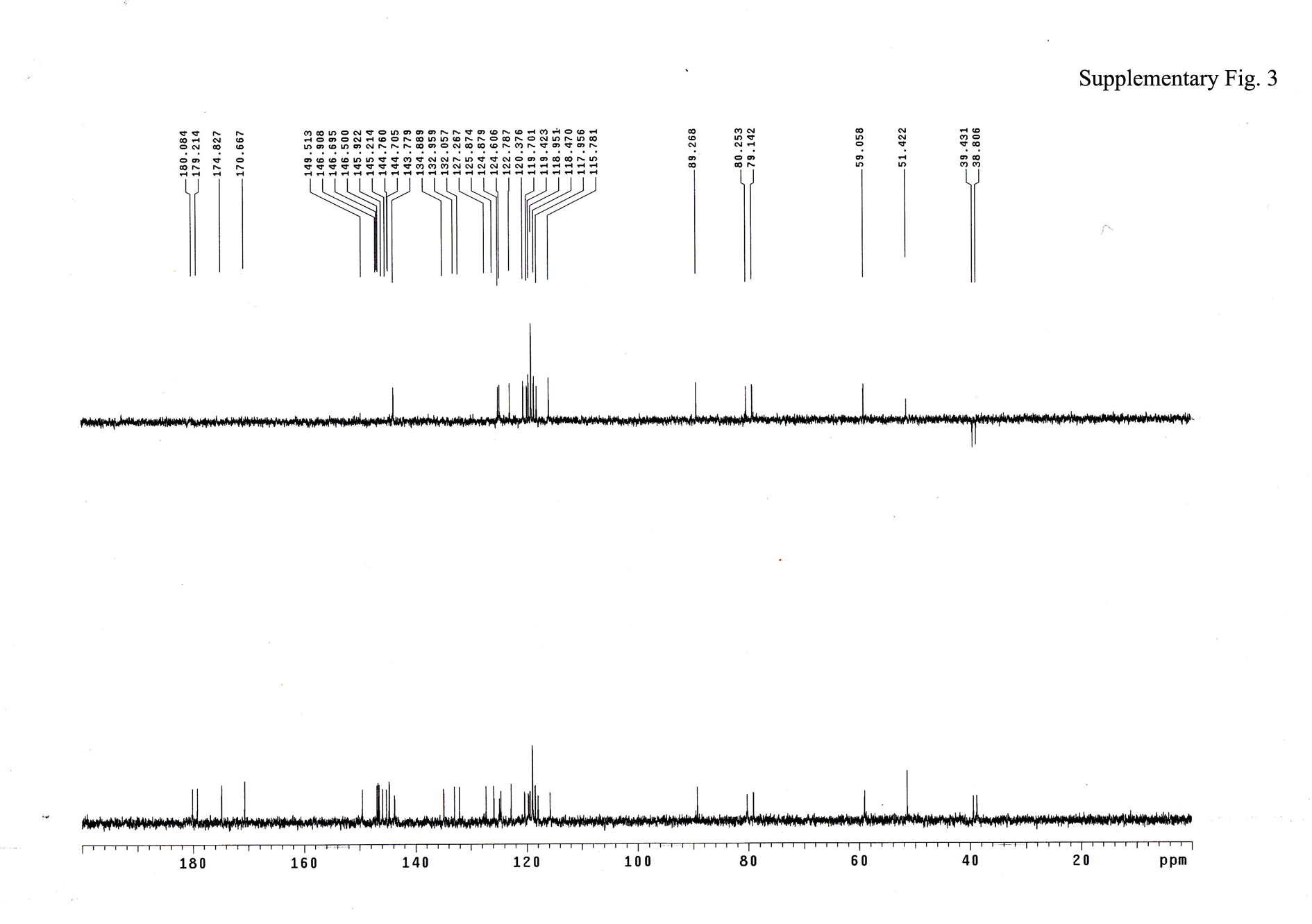

Supplement: Figure S3 — 13C NMR spectrum of SalB. (TIF) [file pone.0024036.s003.tif]

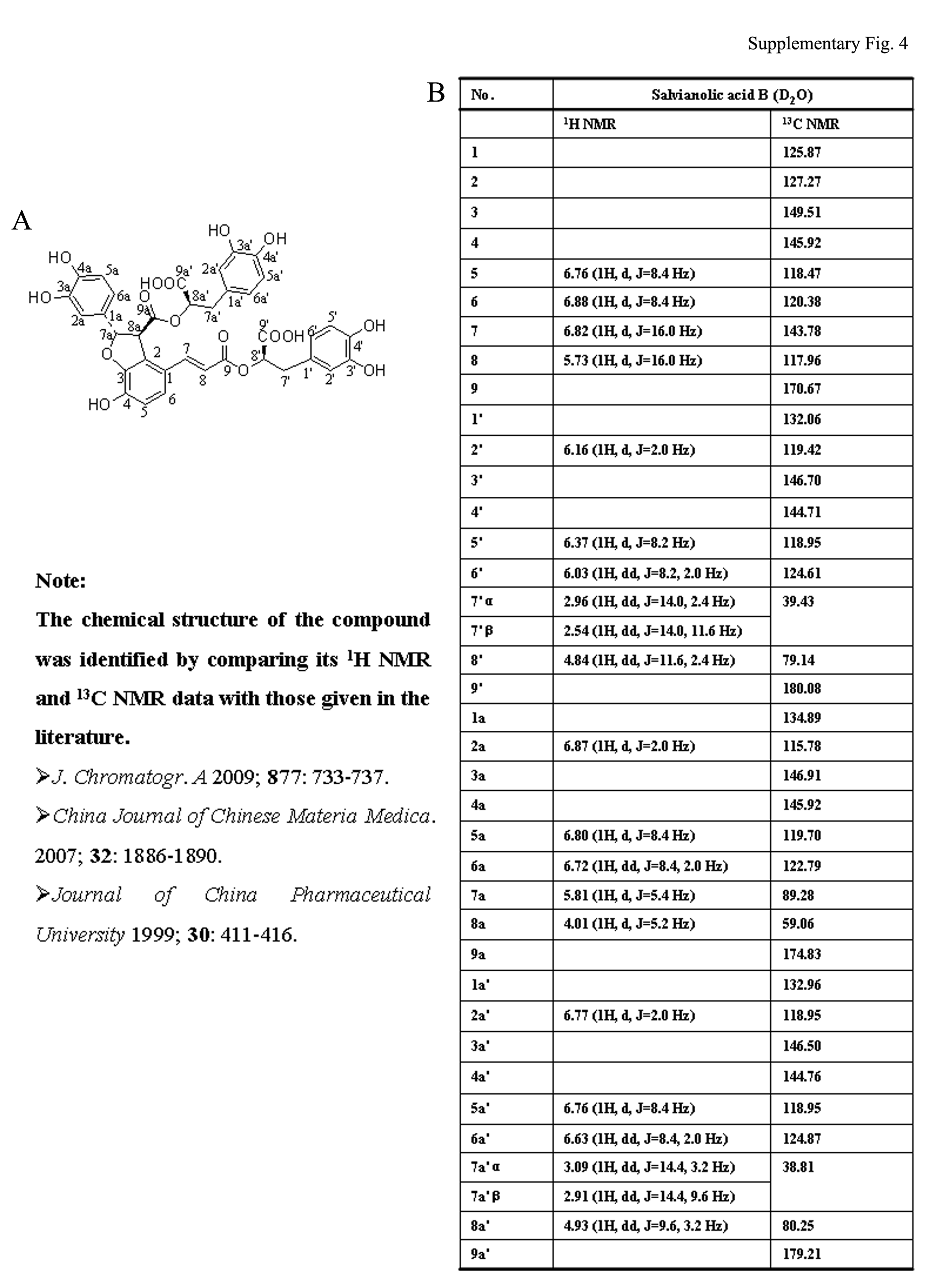

Supplement: Figure S4 — Structure elucidation of SalB. (A) Chemical structure of SalB. (B) 1H NMR (400 MHz) and 13C NMR (100 MHz) spectral data for SalB. (TIF) [file pone.0024036.s004.tif]

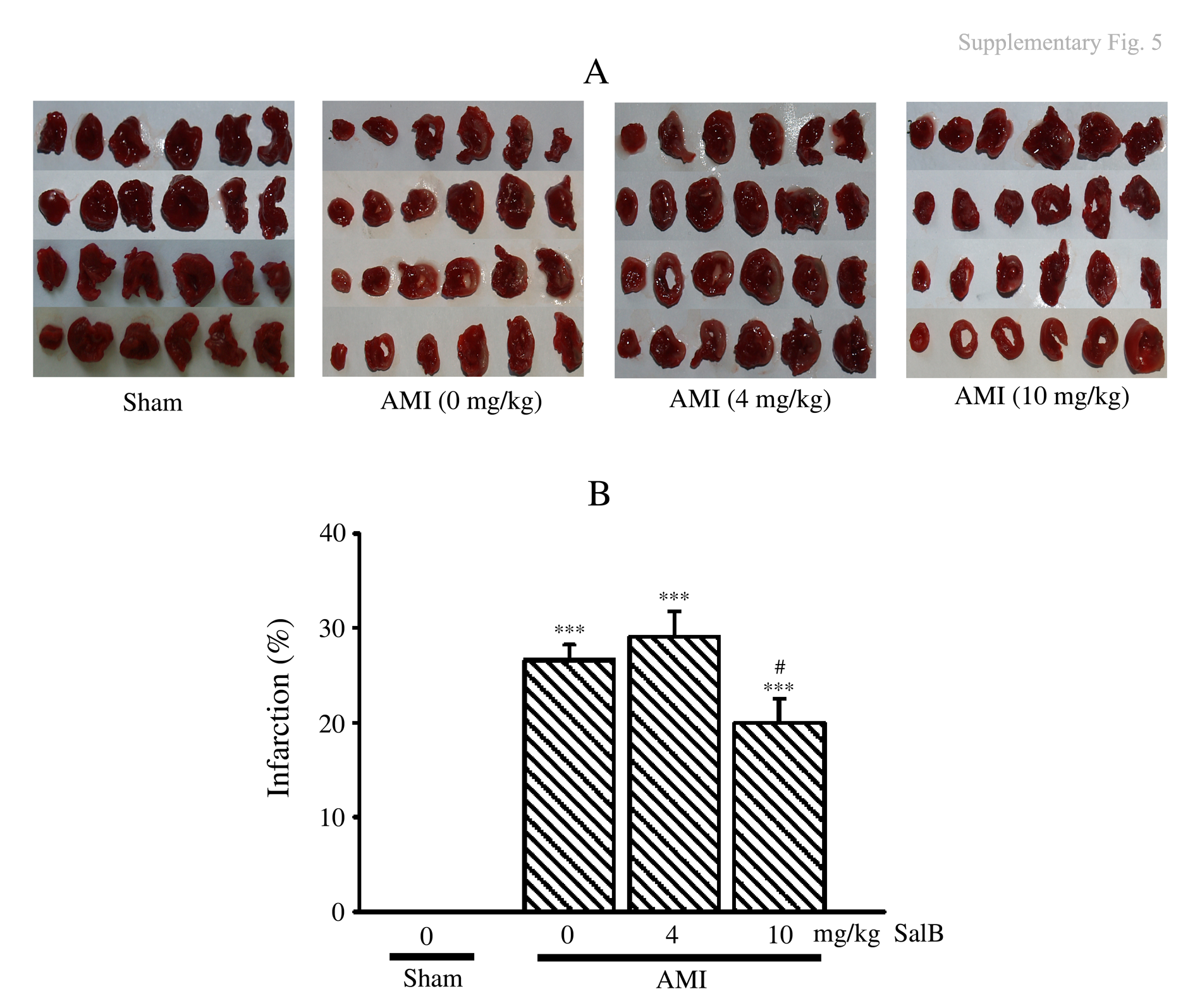

Supplement: Figure S5 — SalB decreased infarct sizes dose dependently. (A) Representative photographs of triphenyltetrazolium chloride stained rat heart after AMI injury. (B) Graphic representation of left ventricle infarct size expressed as percentage of total ischemic area in each group (n = 10). # p<0.05 vs. AMI rats, *** p<0.001 vs. Sham rats. (TIF) [file pone.0024036.s005.tif]
